# The Garbage Enzyme with Chinese Hoenylocust Fruits Showed Better Properties and Application than When Using the Garbage Enzyme Alone

**DOI:** 10.3390/foods10112656

**Published:** 2021-11-02

**Authors:** Sitong Gu, Dongying Xu, Fuhui Zhou, Chen Chen, Chenghui Liu, Mixia Tian, Aili Jiang

**Affiliations:** 1 Key Laboratory of Biotechnology and Bioresources Utilization, Ministry of Education, Dalian 116600, China; gusitong0478@163.com (S.G.); 18840875773@163.com (D.X.); zhoufuhuihui@126.com (F.Z.); chenchen@dlnu.edu.cn (C.C.); liuchenghui@dlnu.edu.cn (C.L.); tmx@dlnu.edu.cn (M.T.); 2 College of Life Sciences, Dalian Minzu University, Dalian 116600, China

**Keywords:** Chinese honeylocust fruits, garbage enzyme, detergent, detoxification agent

## Abstract

Garbage enzyme (GE) is a vinegar or alcohol product derived from fermenting fresh kitchen waste, such as vegetable and fruit residues (peels, cuttings and bits), sugar (brown sugar, jaggery or molasses sugar) and water. Chinese honeylocust fruits (*Gleditsia sinensis*) have been used in China for at least 2000 years as a detergent. The aim of the study was to investigate the properties and application of Chinese honeylocust garbage enzyme (CHGE), which is produced when equal amounts of Chinese honeylocust fruits and fresh wastes are mixed. The results showed that CHGE had lesser microbial communities and lower surface tension than GE. CHGE also had higher viscosity, foam stability and emulsion stability than GE. Compared with GE, CHGE induced higher enzymatic amylase, cellulase, lipase and protease activities. CHGE had stronger detergency than GE and a 100× dilution of CHGE could significantly remove pesticide residues after a 30 min soaking treatment. The study showed that as a biological detergent, CHGE is safer and more environmentally friendly than GE and has remarkable washing and cleaning power. The preparation method of the detergent is simple: it can be prepared at home using fruit and vegetable waste, which is beneficial to the secondary utilization of waste and the reduction of pollution to the environment and damage to human health.

## 1. Introduction

An enormous amount of fruit and vegetable waste is generated in the world due to high production and the lack of effective preservation measures. In addition, with the improvement of the quality of people’s life, as well as the increasing demand for fresh, nutritious and convenient foods, a fresh-cut food industry has emerged and developed very quickly. Fresh-cut products are fruits or vegetables that have been trimmed and/or peeled, or cut into 100% edible products. However, the growing fresh-cut industry has also brought with it large amounts of fruit and vegetable peels and pits, and how to dispose of the fresh cut waste has become a hot topic. The traditional treatments, such as landfill and incineration result in groundwater pollution and the release of carbon dioxide, a greenhouse gas, causing serious environmental pollution and health risks to living organisms. Therefore, alternative environmentally-friendly treatments for fruit and vegetable wastes are urgently needed in order to make use of the wastes and increase the added value of fruit and vegetables as well as minimize the pollution problems created by them. Cleaning products are necessities in our life, and the use of chemical detergents affects human health and causes environmental pollution. Natural-derived products are becoming a new trend in the household products market. Currently, although there are a variety of washing products on the market, including plant extracts and enzyme detergents, they still cannot meet people’s requirements for safety and low price, and are not conducive to the reuse of waste.

Garbage enzyme (GE) is produced by fermentation of waste fruits, vegetables or their peels, sugar (brown sugar or molasses sugar) and water using a method developed by Rosukon in 2006 [1]. It can be a viable method for turning food wastes into a useful resource for the production of higher-value-added products through fermentation processes due to its higher rate of degradation within a shorter period; additionally, the garbage enzyme production cost is cheaper, as it is produced from organic solid waste [2]. It was reported that GE could be used as fertilizer, plant growth hormones, pesticide, insecticide, wastewater treatment and antimicrobial agent, and the functions of GE may be different at different concentrations [3]. Till now, GE research has focused on improving water quality, as well as stabilizing industrial waste activated sludge [4,5,6]. However, to the best of our knowledge no literature to date has been devoted to the scientific study of GE’s components, the effects of its usage and the mechanisms of its reactions with the aim of developing washing products.

Chinese honeylocust (*Gleditsia sinensis* Lam.) is a perennial arbour belonging to the Leguminosae family, which is widely distributed in most parts of the world, especially in China [7]. The most important components of Chinese honeylocust are triterpenoid saponins, triterpenes, flavonoids, alkaloids, phenolics and sterols [8]. Among them, triterpenoid saponins are the principal metabolites, with numerous pharmacological activities, and can be employed in the food and medicine fields [8]. In addition, the plant is a surfactant, and has the advantages of being powerful in activity, having decontaminating and foaming properties, is nontoxic to people and the environment, is easily biodegradable and is cheap, and thus is also used in a wide range of applications in the cosmetics, shampoo and washing industries [9,10]. With the development of the chemical industry, the detergent in Chinese honeylocust was extracted using chemical reactions. However, these chemical treatments waste energy and are not environmentally friendly [11,12].

The analysis of the above cited literature led us to conclude that the garbage enzyme has mainly been used for wastewater treatment, but no in-depth studies were reported on the usage of fruit and vegetable waste as garbage enzyme washing products. Furthermore, the detergent development and efficacy of GE combined with Chinese honeylocust have not been reported to date. Therefore, the purpose of the study was to find an environmentally friendly detergent by using CHGE, in which GE was used to release the active detergent substances from Chinese honeylocust fruits. The microbial communities and their structure in GE and CHGE were studied by high-throughput sequencing. The detergent characteristics were determined, the activities of washing-related enzymes were investigated, and the detoxification and decontamination abilities of CHGE and GE were measured to explore the possible application of CHGE in the washing industry. Meanwhile, the research can also provide reference for household fruit and vegetable waste utilization and homemade detergents.

## 2. Materials and Methods

### 2.1. Preparation of GE and CHGE

**Figure 1:**
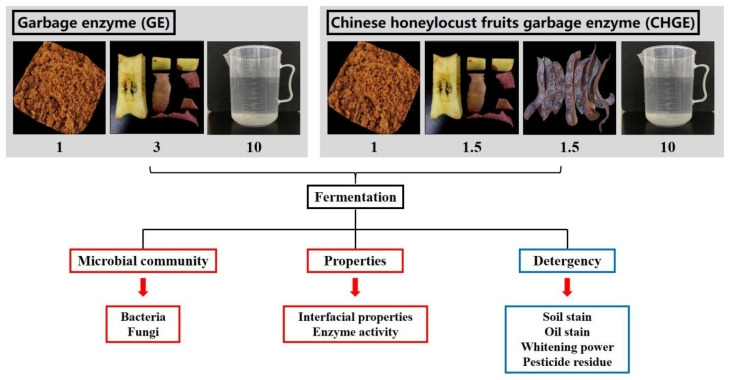
Schematic representation of the experiment workflow.

To make the GE, fresh-cut apple scraps were pulverized using a pulverizer (JYL-G12, Jiuyang Co., Ltd., Jinan, China). One part of brown sugar, three parts of apples particles, and ten parts of water were thoroughly mixed and put in a well-sealed receptacle to ensure airtight conditions [1] (Figure 1). Similarly, to make the CHGE, one part of brown sugar, one and a half parts of fresh apple wastes, one and a half parts of ground Chinese honeylocust fruit powder and ten parts of water were mixed and then placed in sealed receptacles for anaerobic fermentation for three months at room temperature. The fermented materials were filtered three months later, and the liquid was diluted 0-, 10-, 50-, 100-, 200-, and 500-fold for further experiments. The commercial detergent WhiteCat (Shanghai and Huangbai Cat Co., Ltd., Shanghai, China) was diluted 100-fold as a control for further analysis.

### 2.2. High-Throughput Sequencing of the Microbial Communities

High-throughput sequencing was used to identify the microbial communities in CHGE and GE. The DNA from GE and CHGE was extracted using the Fermentation Broth DNA Kit (MoBio Laboratories, Carlsbad, CA, USA) according to the manufacturer’s instructions. A 0.8% agarose gel was used to check the purity and quality of the genomic DNA. Illumina Mi Seq sequencing was used to sequence the purified genomic DNA from GE and CHGE. For bacterial DNA, the primers 338F (ACTCCTACGGGAGGCAGCAG) and 806R (GGACTACHVGGGTWTCTAAT) [13] were used to amplify the V3-4 hypervariable region of bacterial 16S rRNA. The PCR was carried out on a Mastercycler Gradient (Eppendorf, Hamburg, Germany) using the following parameters: initial denaturation at 95 °C for 5 min, followed by 32 cycles of 95 °C for 45 s, 55 °C for 50 s and 72 °C for 45 s, then followed by a final extension at 72 °C for 10 min. The PCR products were purified using a QIAquick Gel Extraction Kit (QIAGEN, Hilden, Germany), quantified using Real Time PCR, and sequenced at Allwegene Company (Beijing, China). For the fungal genomic DNA, the ITS region was amplified using ultra PAGE purified primers (Invitrogen, Carlsbad, California, USA) ITS1F (5-CTTGGTCATTTAGAGGAAGTAA-3) and ITS2 (5-TGCGTTCTTCATCGATGC-3) [14]. The PCR was performed at an initial denaturation at 95 °C for 2 min, followed by 30 cycles of 95 °C for 30 s, 55 °C for 30 s, 72 °C for 30 s and a final extraction at 72 °C for 5 min. Three independent PCR reactions were performed per sample for potentially heterogeneous amplification from the environmental templates. The PCR products were purified using the AXYGEN Gel Extraction Kit (QIAGEN) and quantified using qPCR.

Deep sequencing was performed on the Miseq platform at Allwegene Company. The image analysis, base calling and error estimation were analyzed using Illumina Analysis Pipeline Version 2.6. Richness estimators (Chao1) and diversity indices (Shannon) of each sample were calculated using the software Mothur (version 1.40.0, The University of Michigan, Ann Arbor, MI, USA). To compare the membership and structure of communities in different samples, heat maps were generated with the top 20 OTUs (operational taxonomic unit) using Mothur [15].

### 2.3. Interfacial Properties of GE and CHGE

The surface tension, viscosity, foam stability and solution stability were investigated to compare the characteristic interfacial properties of GE and CHGE. The surface tension of the diluted GE and CHGE was tested using a fully automatic surface tension meter equipped with a platinum pendant (BZY-1, Shanghai Hengping Instrument and Meter Factory, Shanghai, China). The viscosity of GE and CHGE was determined using a rotational viscometer (NDJ-79, Shanghai Changji Geological Instrument Co., Ltd., Shanghai, China). A foam test apparatus (2151 Roche foam tester, Beijing Jinzhiye, Beijing, China) was used to measure foam stability. The foam stability for each solution was recorded by the half-life, which is equivalent to the time required to reduce the initial foam volume by 50%. Conductometric measurement was used to test the stability of GE and CHGE (DDS-307, Shanghai Precision Science Instrument Co., Ltd., Shanghai, China) by recording the voltage potential of the top and the bottom of the solutions [16]. All of the experiments mentioned above were performed at room temperature (25 °C).

### 2.4. Biocatalytic Activity of GE and CHGE

To compare the biocatalytic activity of GE and CHGE, the enzymatic activities of amylase, cellulase, lipase and protease were analyzed.

#### 2.4.1. Amylase Activity

The activity of extracellular amylase was estimated by determining the amount of reducing sugars released from starch, using the 3,5-dinitrosalicylic acid (DNSA) method [17] with some modifications. Four 20 mL plug test tubes were filled with 1 mL of GE or CHGE; then 2 mL of DNSA reagent was added to two of these tubes, followed by incubation at 40 °C for 10 min, and then 1 mL of 1% starch was added to the tubes, which were then incubated at 40 °C for 5 min. Reaction was stopped by the addition of 2 mL DNSA chromogenic reagent, which was boiled for 5 min, to the two remaining test tubes. A total of 16 mL of distilled water was added after the tubes were cooled. The absorbance at 540 nm was read with a spectrophotometer (Shimadzu, Kyoto, Japan) and used to determine the amount of reducing sugars. The maltose was used as a standard. One unit of enzymatic activity was defined as the amount of enzyme that produces 1 mol of reducing sugar as maltose per minute at 540 nm. The amylase activity was calculated as follows:

(1) $$\mathrm{Amylase}\;\mathrm{activity}\;\left[\mathrm{mg}/(\min\cdot g)\right]=\frac{\mathrm{mg}\;\mathrm{of}\;\mathrm{maltose}\;\mathrm{released}\times \mathrm{Dilution}\;\mathrm{factor}}{\mathrm{Volume}\;\mathrm{of}\;\mathrm{solutions}\times \mathrm{Enzymatic}\;\mathrm{reaction}\;\mathrm{time}}$$

#### 2.4.2. Cellulase Activity

The cellulase activity was determined by the standard of the Committee of Biotechnology of the International Union of Pure and Applied Chemistry (IUPAC) [18]. To test the cellulose activity of GE and CHGE, 10 mL of GE or CHGE were added to 100 mL of phosphate buffer solution (PBS) (50 mM, pH = 7.0). A total of 0.5 mL of the GE or CHGE solution was mixed with 2 mL of 1% (*w*/*v*) carboxymethyl cellulose (CMC) dissolved in PBS in 10 mL of colorimetric tubes and incubated at 37 °C for 10 min, followed by quickly adding 2 mL of DNSA, and the tubes were boiled for 10 min for color reaction. Once the reaction was complete, the tubes were cooled with running water and the reaction solution was diluted to 10 mL. The absorbance at 540 nm was read at room temperature with a Shimadzu spectrophotometer. One unit of cellulase activity is a representation of the weight of glucose catalyzed by hydrolysis of CMC at 37 °C per hour.

(2) $$\mathrm{Cellulase}\;\mathrm{acticity}\;(\mu g/h\cdot g)=\frac{\mathrm{mg}\;\mathrm{of}\;\mathrm{glucose}\;\mathrm{released}\times \mathrm{Dilution}\;\mathrm{factor}}{\mathrm{Volume}\;\mathrm{of}\;\mathrm{solutions}\times \mathrm{Enzymatic}\;\mathrm{reaction}\;\mathrm{time}}$$

#### 2.4.3. Lipase Activity

The lipase activity was determined by using the following method [19]. A total of 2.50 mL of ultra-pure water, 1 mL of Tris HCl buffer and 3 mL of olive oil were added to conical flasks and 1 mL of GE or CHGE was added to the flasks. The GE or CHGE solutions were mixed, followed by a 15 min incubation at 37 °C, and then 3 mL of 95% ethanol solution and 3–4 drops of phenolphthalein indicator were added to the reaction mixtures. The final reactions were titrated with NaOH until a light pink color appeared. One unit of lipase activity was expressed as the amount of enzyme that releases 1 mol of fatty acids per minute.

(3) $$\mathrm{Lipase}\;\mathrm{activity}\;(\mu /g)=\frac{(\mathrm{Volume}\;\mathrm{of}\;\mathrm{NaOH}\;\mathrm{used}\;\mathrm{for}\;\mathrm{test}-\mathrm{Volume}\;\mathrm{of}\;\mathrm{NaOH}\;\mathrm{used}\;\mathrm{for}\;\mathrm{blank})\times \mathrm{Dilution}\;\mathrm{factor}}{\mathrm{Volume}\;\mathrm{of}\;\mathrm{solution}}$$

#### 2.4.4. Protease Activity

A total of 1 mL of GE or CHGE was added to 2.5 mL 1% (*w*/*v*) casein solution dissolved in 50 mM PBS (pH 7.5). A total of 2.5 mL 0.4 M trichloroacetic acid solution was added into the mixture after incubation at 40 °C for 10 min. Then, 1 mL of 3× diluted Folin reagent was added to the reaction. The absorbance values were recorded at 660 nm with a spectrophotometer (Shimadzu) after incubation at 40 °C for 20 min. Protease activity was defined as the amount of enzyme that releases 1 μg tyrosine per gram of plant per minute [20].

(4) $$\mathrm{Protease}\;\mathrm{activity}\;(\mu /g)=\frac{\mathrm{mg}\;\mathrm{of}\;\mathrm{tyrosine}\;\mathrm{released}\times \mathrm{Dilution}\;\mathrm{factor}}{\mathrm{Volume}\;\mathrm{of}\;\mathrm{solutions}\times \mathrm{Enzymatic}\;\mathrm{reaction}\;\mathrm{time}}$$

### 2.5. Test of Detergency

The detergency of GE and CHGE was tested by using an ultrasonic cleaner (KQ5200DB, Kunshan Ultrasonic Instrument Co., Ltd., Kunshan, China) for the decontamination of fabrics treated with tillage soil and cooking oil at room temperature. A total of 20 mL of different dilutions of GE and CHGE was used for washing of the fabrics. Clean water was used as the negative control and commercial detergent was used as the positive control. The stirring type was automatic mode: power supply: 220 V~/50 Hz; rated washing input power: 345 W; weight of washing: 4.6–5.5 kg; energy efficiency rating: secondary; washing time: 20 min; drying for 5 min. Unwoven fabric was cut into sizes of 1 m × 1 m, and nine pieces were used for each test. After washing, the cleaning effects of GE and CHGE were assessed by determining the whiteness of each fabric with a Chroma Meter (CR-400, Konica Minolta Optics, Inc., Tokyo, Japan). The detergency was calculated using the following Equation (5) [16]:

(5) $$\mathrm{Detergency}\;(\%)=\left[\frac{C-B}{A-B}\right]\times 100$$

where A is the whiteness of the white cloth, B is the whiteness of the dirty cloth before washing, and C is the whiteness of the dirty cloth after washing.

### 2.6. Detoxification Ability of GE and CHGE

Pak-choi treated with pesticides was used to test the detoxification ability of GE and CHGE. The pak-choi was sprayed with a 1000× dilution of dichlorvos and chlorpyrifos repeatedly and evenly, and then the treated pak-choi was put in a ventilated area for 12 h. Amounts of 5 g of pak-choi treated with the pesticides were soaked in GE or CHGE for 20 min, 30 min and 40 min. The pak-choi was taken out and put into a beaker containing 10 mL of PBS (pH 8.0); 2.5 mL of the supernatant was taken out after 2 min of shaking, while 2.5 mL of PBS treated solution was used as control.

A total of 0.1 mL of acetylcholinesterase was mixed with the supernatant for 10 min, and then 0.1 mL of dithiodinitrobenzoic acid and sodium hydrogen carbonate (a chromogenic reagent) and 0.1 mL of thioacetylcholine were added into the reaction. The enzyme inhibition rate was measured at 410 nm and the removal rate was calculated using the following Equation (6) [21]:

(6) $$\mathrm{Inhibition}\;\mathrm{rate}\;(\%)=\frac{\Delta \mathrm{Ac}-\Delta \mathrm{As}}{\Delta \mathrm{Ac}}\times 100$$

where ΔAc is the change value of the absorbance of the control solution after 3 min, and ΔAs is the change value of the absorbance of the sample solution after 3 min:

(7) $$\mathrm{Removal}\;\mathrm{rate}\;(\%)=\frac{\mathrm{Rc}-\mathrm{Rt}}{\mathrm{Rc}}\times 100$$

where Rc is the inhibition rate of enzymes in the untreated sample, and Rt is the inhibition rate of enzymes in the treated sample.

### 2.7. Statistical Analysis

SPSS version 17.0 (SPSS Inc., Chicago, IL, USA) was used for all statistical analyses. Differences between GE and CHGE were determined using a one-way ANOVA and the means were compared using LSD at *p* < 0.05.

## 3. Results

### 3.1. Analysis of the Diversity of the Microbial Community in GE and CHGE

High-throughput sequencing of bacteria and fungi was performed to determine the microbial community of GE and CHGE. Good’s coverage of all the samples ranged from 99.43 to 99.95%, indicating that the sequencing was reliable when identifying the majority of diversity in the GE and CHGE samples. In terms of the quantity of operational taxonomic unit (OTU), GE had 456 OTUs for bacteria and 133 OTUs for fungi, indicating that GE had a richer diversity of bacterial and fungal communities than that of CHGE, which had 310 OTUs for bacteria and 84 OTUs for fungi (Table 1). The values of Shannon and Chao 1 revealed that GE had more bacterial and fungal diversity than that of CHGE, further indicating that bacteria and fungi in the GE are richer than that in CHGE.

**Figure 2:**
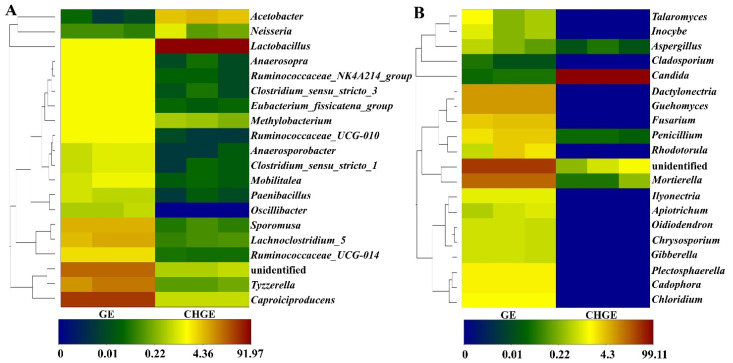
Heatmap clustering analysis of relative abundance of the top 20 communities for dominant bacterial genus (**A**) and fungal genus (**B**) in GE and CHGE.

Figure 2A shows the top 20 bacterial genera that showed higher relative abundance in GE and CHGE. The dominant genera present in GE were *Caproiciproducens*, an unidentified species, *Tyzzerella*, *Sporomusa*, and *Lachnoclostridium_5*, while *Lactobacillus* had the highest abundance in CHGE. The difference in relative abundance of genera between GE and CHGE was significant.

The differences of fungal community at the genus level are shown in Figure 2B. There were seven dominant genera in GE with a relative abundance of >0.1%: an unidentified species, *Mortierella*, *Dactylonectria*, *Guehomyces*, *Fusarium*, *Penicillium*, and *Rhodotorula*. *Candida* were the only dominant genera present in CHGE. This experiment showed that the Chinese honeylocust fruits could significantly reduce the bacteria and fungi community of GE.

### 3.2. Interfacial Properties of GE and CHGE

To characterize the interfacial property of GE and CHGE, the surface tension, viscosity, foam stability and solution stability of these detergents were investigated.

The pH value increased with the dilutions of GE and CHGE, but there was no difference between GE and CHGE at all dilutions (Table 2). The pH of the commercial detergent was 1.41 and 1.29 times that of GE and CHGE at 100 times dilution, respectively.

The surface tension increased with the dilution times for both GE and CHGE. The surface tension of CHGE was smaller than that of GE at all dilutions (Table 2). The water had highest surface tension (44.2 nM/m) and WhiteCat, the commercial detergent, had the lowest surface tension (23.8 nM/m) (Table 2).

The viscosity of GE and CHGE decreased with the increase of the dilution times (Table 2). The commercial detergent WhiteCat had the highest viscosity of 16.2 mPa·s, while both the undiluted detergents had a value of 14.3 mPa·s for GE and 14.1 mPa·s for CHGE. No significant differences were found between GE and CHGE at all dilutions (Table 2).

The half-life of the foam (the time needed for half of the foam to disappear) was recorded to test the foam stability. Foam stability was consistent in both GE and CHGE, and the half-life was gradually shortened with the increase of the dilution times. Interestingly, the half-life of CHGE was longer than that of GE at all dilutions. The commercial detergent WhiteCat had the best foam stability, with a half-life of 2005 s, while the water, as expected, showed no foam (Table 2).

The emulsion stability of GE and CHGE was estimated by comparing the conductivity values of GE and CHGE. Except for undiluted GE and CHGE, the emulsion stability was considered to be stable for both GE and CHGE at all dilutions because there was no significant difference between the conductivity values for the top and bottom of these solutions (Table 2).

### 3.3. The Activities of Washing-Related Enzymes in GE and CHGE

**Figure 3:**
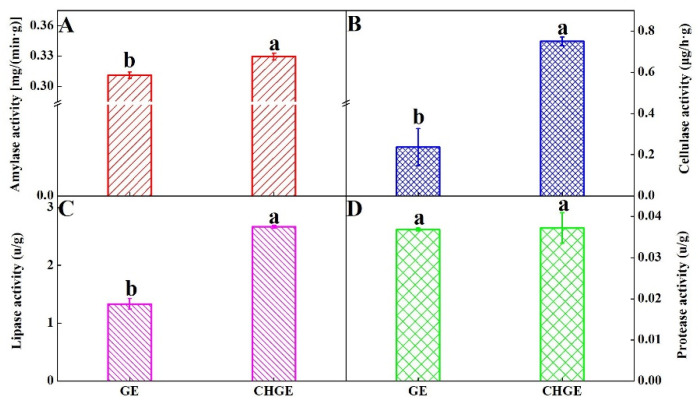
Activities of amylase (**A**), cellulase (**B**), lipase (**C**), and protease (**D**) of GE and CHGE. Data represent the mean ± SD (*n* = 3). Different letters indicate significant differences (*p* < 0.05) between GE and CHGE.

The use of enzymes in detergent formulations is now common, with over half of all detergents presently available containing enzymes. The activity of four washing-related enzymes were compared in GE and CHGE. The results showed that the activities of amylase, cellulose and lipase of CHGE were significantly higher than that of GE (*p* < 0.05) (Figure 3A–C). However, no difference was found for protease activity between GE and CHGE (Figure 3D). It should be noted that the cellulase of CHGE was three times that of GE and the lipase activity of CHGE was 2.2 times that of GE (Figure 3B,C).

### 3.4. Decontamination Ability of GE and CHGE

**Figure 4:**
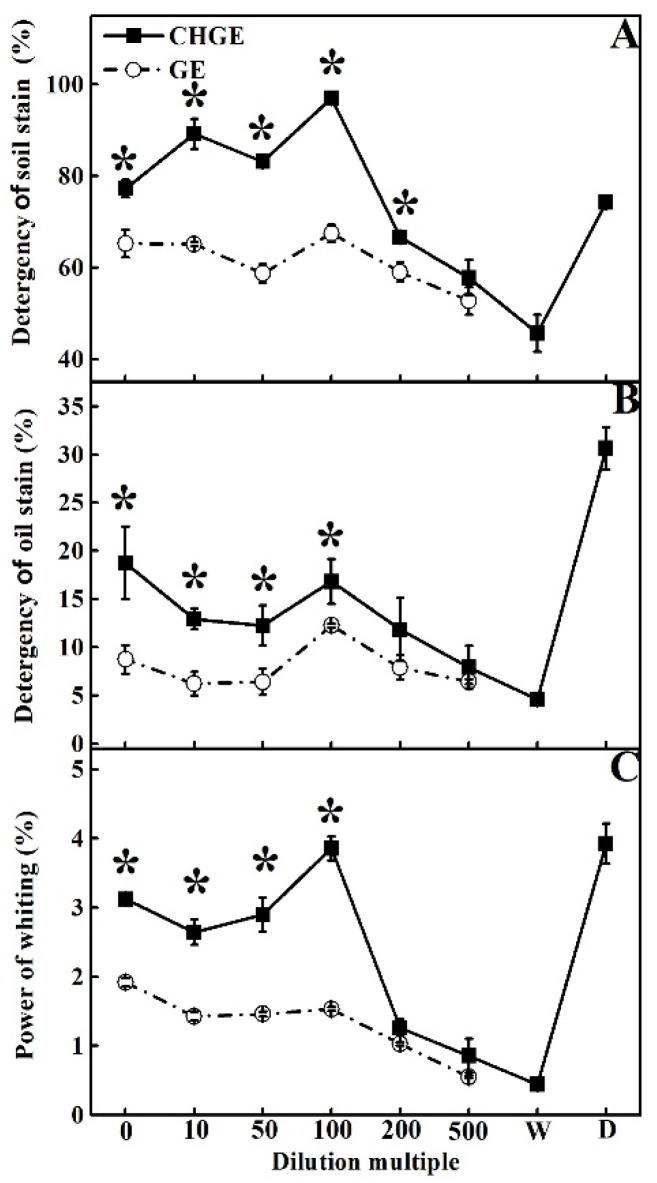
Effects of GE and CHGE on detergency of soil stains (**A**), oil stains (**B**), and whitening power (**C**). Data represent the mean ± SD (*n* = 3). Asterisks (*) indicate significant differences (*p* < 0.05) between GE and CHGE. D: commercial detergent (WhiteCat, Shanghai and Huangbai Cat Co., Ltd., Shanghai, China), W: deionized water.

Tillage soil and cooking oil were used to contaminate fabric samples and then GE and CHGE were used as detergents to wash them. With the increase of dilution times, the decontamination ability of the two garbage enzyme types on the soil stain both increased and reached the highest detergency at 100-fold dilution, and then decreased with the dilution times (Figure 4A). The detergency of CHGE at 0–200-fold dilutions was significantly higher than that of GE (*p* < 0.05) (Figure 4A). It is worth mentioning that CHGE at 0–100 times dilutions showed better ability in removing of the soil stains than the commercial detergent WhiteCat, and its soil stains removal power was 1.44 and 1.31 times than that of GE and WhiteCat, respectively (Figure 4A).

For the detergency of the oil, the decontamination abilities of GE and CHGE had a similar tread to that for soil. The detergency of CHGE was higher at all dilutions and 0–100 times dilutions was significantly higher than that of GE (*p* < 0.05) (Figure 4B). The commercial detergent WhiteCat had the best detergency for the oil stains (Figure 4B).

Both GE and CHGE showed whitening power on the fabrics (Figure 4C). The whitening power of CHGE was significantly higher than that of GE at 0–100-fold dilutions (*p* < 0.05). The best whitening value for CHGE was 3.86% at 100-fold dilution, only slightly lower than that of the commercial detergent WhiteCat. However, the 500-fold dilution of GE had the weakest whitening power—0.55%, only 0.11 times higher than that of water (Figure 4C).

### 3.5. Detoxification of GE and CHGE

**Figure 5:**
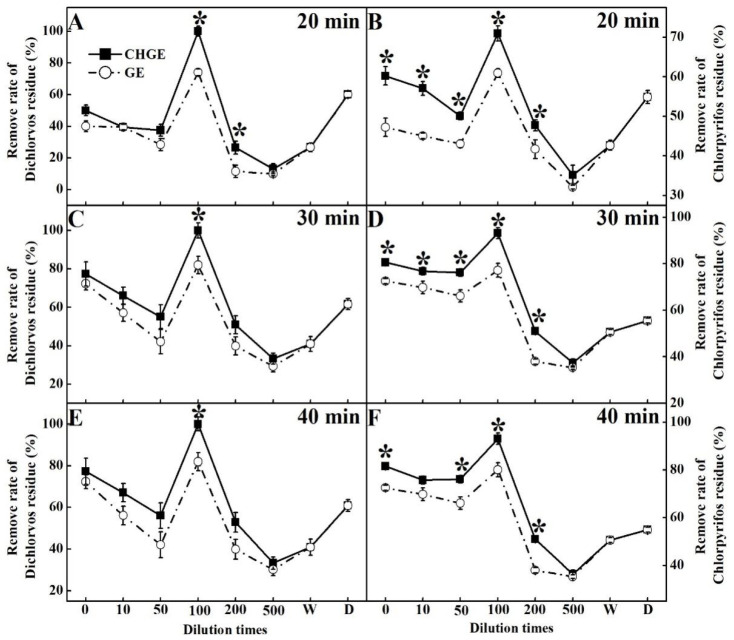
Effects of GE and CHGE on the removal rates of Dichlorvos and Chlorpyrifos residue after soaking the pak-choi for 20 min (**A**,**B**), 30 min (**C**,**D**), and 40 min (**E**,**F**). Data represent the mean ± SD (*n* = 3). Asterisks (*) indicate significant differences (*p* < 0.05) between GE and CHGE. D: commercial detergent (WhiteCat, Shanghai and Huangbai Cat Co., Ltd., Shanghai, China), W: deionized water.

GE and CHGE were used as detoxification agents to wash the pak-choi treated with pesticides. Both CHGE and GE removed pesticides at all dilutions, and CHGE had higher removal rates than those of GE (Figure 5). Interestingly, both GE and CHGE had the best removal rate for dichlorvos residues and chlorpyrifos residues when diluted 100 times, even surpassing the ability of the commercial detergent (Figure 5). In terms of the soaking time, 30 min and 40 min soaking were better than 20 min, but no significant difference between 30 min and 40 min treatments was found (Figure 5). After soaking for 40 min, the removal rate of dichlorvos residue in CHGE was 21.97% and 64.20% higher than that in GE and the commercial detergent, respectively, and the removal rate of chlorpyrifos residues was 16.22% and 69.64% higher than that in GE and the commercial detergent, respectively.

## 4. Discussion

Restaurants, vegetable and fruit markets and food processing industries generate waste such as fruits, vegetables and their peels in huge quantities, which not only causes environmental pollution, but also threatens people’s health. In the current study, fruit and vegetable waste and Chinese honeylocust were combined to produce a new detergent—CHGE—which can effectively treat the waste and improve the product value of the fruit and vegetable industry, and also ensure the safety of the detergent. The results indicated that CHGE contained significantly reduced microbial species. Most importantly, the microorganisms in CHGE are non-pathogenic, indicating the safety of CHGE as a detergent. The research also indicated that CHGE has better interfacial properties and higher enzymatic activity of the washing-related enzymes than that of GE, which led it to an efficient detergent. Additionally, the CHGE at 100-fold dilutions had higher detergency levels of soil stains and oil stains and higher whitening power than those of GE and the commercial detergent, and could also effectively remove pesticide residues; thus, it is a better detoxification agent than GE and the commercial detergent. The results of this study may contribute to the development of consumer products that are of natural origin, environmentally friendly and safe for human health.

The production process of GE is oxidation in the absence of air or through natural fermentation; the fermentation process and the acidic environment provide for the extraction of enzymes from the waste being fermented, and the acids and alcohols produced during fermentation make the GE an efficient anti-microbial agent [22]. It has been reported that Chinese honeylocust possesses anti-microbial, antibacterial and antifungal qualities [8]. Our results indicated that CHGE significantly reduced the species and relative abundance of bacterial genera and fungal genera compared with GE, which could be due to the addition of Chinese honeylocust, which enhanced the inhibition effect of the growth of microorganisms [23]. On the other hand, Chinese honeylocust is an alkaline substance [24] and can weaken the acidity of the GE; our research also suggested the pH value of CHGE was higher than that of GE, which could inhibit the growth of acidophilic bacteria and is safer than GE. Arun et al. [6] tested the antimicrobial activity of GE produced by fermentation and found that when the pH of the GE increased from 3.6 to 7 the activity of extracellular enzymes present in the GE solution increased, which in turn enhanced the antimicrobial activity and confirmed that the GE has a pathogen killing/inhibiting property. It is worth noting that *Lactobacillus* is a type of probiotic bacteria that can improve fermentation capacity; the relative abundance of *Lactobacillus* is higher in CHGE than in GE, while candida was the only dominant fungal genus presented in CHGE. These phenomena may be related to the addition of Chinese honeylocust, which is more suitable for the living environment of lactobacillus and candida. The results also indicated that CHGE is a safer detergent.

A foam is a complex dispersion of bubbles or gases in a liquid or solid matrix [25]. Foams are a common attribute of detergent products, and the surface tension and viscosity are related to form formation [26]. The aqueous phase bubbles consist of air bubbles in the water that are metastable and need to be stabilized by surface active molecules [27]. In many industrial and commercial applications, e.g., enhanced oil recovery, mineral flotation, food products, personal care products and detergents, the generation of foams as well as the foam stability are important [28]. Saponins can decontaminate and foam and have been extensively used in detergents, with high economic value [29]. However, in detergent applications, this does not mean the more the foam, the better the effect, because too much foam will increase unnecessary washing time and will waste water. Shendel et al. [30] also found the height of foam in distilled water produced by garbage enzyme to be lower than in commercial washing products, such as Nirma, Tide and Rin. On the other hand, the emulsion stability of GE and CHGE was determined by electrical conductivities on the top and bottom. In this study, foam stability was expressed by the half-life of the bubbles. The longer the half-life is, the more stable the foam is. The CHGE had longer half-lives than GE while little difference was found in conductivities when compared with GE, indicating that the CHGE is a better detergent than GE. Our results are consistent with the study of Do et al. [10], who indicated that the higher the concentration of saponin in the formulation, the higher the values of foam volume observed 10 s after its formation.

Enzymatic detergents are an emerging type of effective detergents, which now occupy a place of eminence among varied types of laundry detergents, and protease, lipase, amylase, and cellulase are the most commonly used enzymes in these formulations. Amylase is the most important enzyme in the washing industry and several studies have shown that amylase could degrade the residue of starchy foods [31]. Cellulase has a role in color-reviving agents in detergent [32]. Lipase adsorbs on the surface of the fabric to form a stable fabric–lipase compound, and hydrolyzes oil stains on the fabric during the washing [33]. Enzymatic detergents, even when they contain only very small amounts of enzymes, have significantly better washing effects than those who do not use enzymes. Enzyme products have been widely used as detergent builders in recent years because of their lower toxicity, non-corrosiveness, environmental friendliness, excellent biodegradability and increased improvement properties [34,35]. It has been reported that garbage enzymes produced from preconsumer organic waste contain multihydrolytic enzyme activity, which helps solubilize waste-activated sludge [36]. Amin et al. [2] reported that the maximum activity of lipase was found after 96 h of reaction in a fermentation medium with initial pH 4 using agricultural waste as a raw material. The present study showed that CHGE had higher activities of amylase and cellulase than those of the GE group, which explains why CHGE had higher soil stain detergency and whitening power, and the detergency of soil stains was more effective than with commercial detergent, which agrees with the findings of Singh et al. [35] and Ladeira et al. [37]. CHGE also showed stronger detergency for oil stains, which can probably be attributed to its higher lipase activity. CHGE contains natural enzymes with higher activity and thus has stronger detergency ability.

Increasing pesticide use in recent years has led to public concern about the impacts of pesticide residues on human health and in the environment [38]. In the 21st century, the use of organic synthetic pesticides has increased about 40-fold, while organophosphorus pesticides account for 20–38% of all pesticides all over the world [39], of which dichlorvos (2,2-dichlorovinyl dimethyl phosphate, DDVP) and chlorpyrifos (CHP, O,O-diethyl-O-[3,5,6-trichloro-2-pyridinyl], C_9_H_11_C_l3_NO_3_PS) are the most commonly used pesticides from the organophosphorus pesticides family. CHP is an irreversible inhibitor of cholinesterase, and is a most harmful pesticide to all animal species and humans [40]. As a chlorinated organophosphorus insecticide, DDVP is extremely toxic to humans. Acute exposure to DDVP can cause breathing problems, coma or even death [41]. It is very important to remove the residues of the pesticides in washing. Enzymatic bioremediation is potentially a rapid method of removing environmental pesticide residues [38]. Our study showed that both GE and CHGE could effectively remove CHP and DDVP sprayed on pak-choi, and 100-fold dilution of CHGE had the best effect in removing the pesticides, probably due to the lower surface tension of CHGE. Surface tension is the force between molecules, which decreases the surface area of a liquid [42]. It is believed that surface tension increases with a graduate increase in the hydrophilicity of a surfactant [43]. With the higher surface tension, CHGE has a stronger ability to remove pesticide residues at all dilutions. This could be due to the fact that the triterpenoid saponin contained in Chinese honeylocust fruits is a surfactant, which could reduce surface tension [44]. As is known, the main component of commercial detergents is a surfactant, which explains its surface tension was lower than other treatments even the detergent was diluted.

## 5. Conclusions

In conclusion, CHGE is a safe detergent because it has a relatively small microbial community and contains no pathogenic pathogens. CHGE at 100-fold dilution had the best washing ability based on the results of detergent characterization, enzyme activity and detergency. CHGE is also a potential detoxification detergent. Therefore, CHGE detergent is conducive to the secondary use of fruit and vegetable waste and the reduction of environmental pollution; additionally, it can be made at home with the characteristics of safety, low cost, convenience and pollution reduction, and can replace some commercially available washing products. In future work plan to continue to improve the cleaning efficacy of CHGE detergent, and prepare multi-functional and high-efficiency CHGE detergents to enhance commercial competitiveness.

## Figures and Tables

**Table 1:** OTUs, Good’s coverage, and Chaol’s and Shannon’s index for high-throughput sequencing of 16S rRNA and 18S rRNA sequencing of the bacteria and fungi in GE and CHGE.

Sample ID	OTU	Good’s Coverage	Chaol	Shannon
GE_Bacteria	456	99.58%	275.19	4.74
CHGE_Bacteria	310	99.49%	258.41	1.95
GE_Fungi	133	99.83%	242.89	4.17
CHGE_Fungi	84	99.95%	46.84	0.25

**Table 2:** Summary of interfacial properties of GE and CHGE at different dilutions at 25 °C.

Sample	Dilution Times	pH	Surface Tension (mN/m)	Viscosity (mPa·s)	Foam Stability (s)	Emulsion Stability (μs/cm)		
						Top	Bottom	ΔE
GE	0	3.5	32.8	14.3	387	577.0	600.0	23.0
	10	3.7	35.5	11.4	108	114.4	115.2	0.8
	50	3.9	37.3	9.9	35	60.3	60.8	0.5
	100	4.1	38.5	9.1	20	45.1	45.6	0.5
	200	4.2	41.3	9.0	The foam disappears after 3–4 s	33.0	33.1	0.1
	500	4.4	41.9	7.7	The foam disappears after 1–2 s	21.6	21.7	0.1
CHGE	0	3.7	28.6	14.1	400	576.0	590.0	14.0
	10	3.8	30.8	11.9	123	114.9	115.4	0.5
	50	4.0	31.4	10.3	38	60.1	60.6	0.5
	100	4.5	32.6	9.7	21	45.3	45.6	0.3
	200	4.8	38.2	9.2	The foam disappears after 3–4 s	32.7	32.9	0.2
	500	5.1	40.7	7.9	The foam disappears after 1–2 s	21.3	21.6	0.3
White Cat	100	5.8	23.8	16.2	2005	670.0	687.0	17.0
Water		6.8	44.2	1.0	No foam appears	1.2	1.3	0.1

## Data Availability

Not applicable.
